# The Vascularised Groin Chamber: A Novel Model for Growing Primary Human Liposarcoma in Nude Mice

**DOI:** 10.4021/wjon496w

**Published:** 2012-04-23

**Authors:** Daniel Johannes Tilkorn, Sammy Al-Benna, Joerg Hauser, Andrej Ring, Lars Steinstraesser, Adrien Daigeler, Inge Schmitz, Hans Ulrich Steinau, Ingo Stricker

**Affiliations:** a Operative Reference Centre for Soft Tissue Sarcoma, Department of Plastic Surgery, BG University Hospital Bergmannsheil, Ruhr University Bochum, Buerkle-de-la-Camp-Platz 1, 44789 Bochum, North Rhine-Westphalia, Germany; b Institute of Pathology, BG University Hospital Bergmannsheil, Ruhr University Bochum, Buerkle-de-la-Camp-Platz 1, 44789 Bochum, North Rhine-Westphalia, Germany

**Keywords:** Angiogenesis, Soft tissue sarcoma, Tissue engineering, Xenograft

## Abstract

**Background:**

The preclinical development of anti-sarcoma drugs has been primarily based on the subcutaneous transplantation of xenografts. Transplant survival remains an obstacle of current models which has been attributed to the period of hypoxia after transplantation. We hypothesized that primary soft tissue sarcoma models with an intrinsic tissue engineered vascular supply would be easily reproducible. The aim of this study was to establish a model of primary human soft tissue sarcoma with an intrinsic vascular supply.

**Methods:**

Primary soft tissue sarcoma cells from resected human liposarcomas isolated and divided into tumour fragments were transplanted into a silicon chamber, placed around the superficial epigastric vessels in mice. Sarcoma xenograft samples were analysed histomorphologically (light/electron microscopy and immunohistochemistry).

**Results:**

All primary soft tissue sarcoma transplants engrafted, leading to solid tumours within 3 weeks. Histological and immunohistochemical staining confirmed the mouse xenografts as identical high grade liposarcomas compared to original tumour tissue.

**Conclusion:**

This study established a reproducible xenograft model of primary human liposarcoma. This animal model could be of high value for studying human soft tissue sarcomas and their therapy.

## Introduction

Liposarcomas are tumors derived from primitive mesenchymal cells that undergo adipose differentiation and constitute approximately 10% of all soft tissue sarcomas. Liposarcomas are predominantly a disease of adulthood with peak incidence around the 5th to 6th decade of life and with a slight predominance toward males. The extremities are the most common primary site, accounting for about 40% of cases. Nearly one-third of cases arise in visceral spaces, and there is an association between site of primary tumor and histologic subtypes [1]. Histologic grade is one of the most important predictors of outcome, with low-grade myxoid tumours having significantly better survival rates compared to the round-cell, pleomorphic, and dedifferentiated subtypes [2-5]. Complete surgical resection remains the mainstay of local therapy, but adjuvant radiation therapy is effective at controlling microscopic residual disease after surgical resection [6-10]. Local tumour recurrence and the development of distant metastases mainly into the lungs remain unsolved clinical problems in the treatment of soft tissue sarcomas [5]. Metastatic disease becomes evident within the first 2 - 3 years after initial diagnosis and is the main cause of mortality in these patients [11]. The use of chemotherapy for treatment of liposarcoma is controversial, but myxoid tumors appear to respond better than dedifferentiated or well-differentiated tumors [3, 7, 12]. Despite an aggressive multi disciplinary treatment (surgery, chemotherapy and radiation therapy), the rate of recurrence of more than 50% remains very high and results in diffuse metastatic disease and the death of the patients [13]. Treatment for sarcomas has lagged behind more common epithelial cancers, and survival from the high grade liposarcomas has remained unchanged for several decades [14, 15]. The mechanisms associated with sarcoma development remain largely unclear because of the rarity of the disease, its large number of histological subtypes and its varied clinical behaviour [14, 15]. As such, preclinical models to dissect mechanisms underlying sarcoma development, progression and treatment are greatly needed [14, 15].

Subcutaneous and orthotopic xenografts of human tumours in nude mice are an accepted model for *in vivo* biological and preclinical studies [14-19]. Xenotransplantation has become a widely used tool both to demonstrate the tumourigenicity of cells and to test the efficacy of therapeutic interventions *in vivo* [20]. The stability and comparability regarding phenotype, differentiation and characteristics of malignancy are important prerequisites of any tumour model. The subcutaneous implantation of tumour fragments into immunocompromised nude mice is a widely accepted model for the study of various tumours [14, 15, 17, 19, 21] but there are limited primary sarcoma models due to difficulties in establishing reproducible xenograft models of primary human soft tissue sarcoma [17, 19, 21]. We hypothesized that hypoxic conditions after transplantation may in part be responsible for the cytological instability of the xenotransplants. We hypothesized that primary soft tissue sarcoma models with an intrinsic tissue engineered vascular supply would be easily reproducible. The aim of this study was to establish a reproducible xenograft model of primary human soft tissue sarcoma with an intrinsic tissue engineered vascular supply in nude mice.

## Materials and Methods

### Animals

Six weeks old, sexually mature NMR nude mice (Harlan Winkelmann GmbH, Borchen, Germany) weighing about 20 - 25 g were used in the present study. Animals were housed in ventilated racks with controlled humidity and temperature (20 ± 2 °C) under pathogen-free conditions and a 12 h light-dark photoperiodicity and. Food and water as well as boxes, bedding, were sterilised. The surgical procedures used for the inoculation of the cells to give rise to solid tumours and for subsequent removal and transplantation of tumours was conducted under general anaesthesia and steril conditions. All experimental procedures were carried out under the guidelines and with the permission of the ethics committee of the Ruhr University Bochum. Animal care and manipulation adhered to institutional guidelines and the Guide for the Care and Use of Laboratory Animals [22]. Written informed consent of the patient and the permission of the ethics committee of the Ruhr University of Bochum were obtained for the use of tumour specimens.

### Isolation of sarcoma specimens for xenotransplantation

On the day of surgery soft tissue sarcoma samples were directly transferred from the operating theatres to the laboratory. Following the tumour ablation tumour samples were taken under sterile conditions at a representative area of the original tumour mass. The sample was then divided into two adjacent parts, one for further histology (conventional light/electron microscopy and immunohistochemistry) and the other for the *in vivo* experiment. The tumour tissue was cut into 1 - 2 mm fragments prior to transplantation.

### Vascularised groin chamber and *in vivo* sarcoma xenograft model

**Figure 1:**
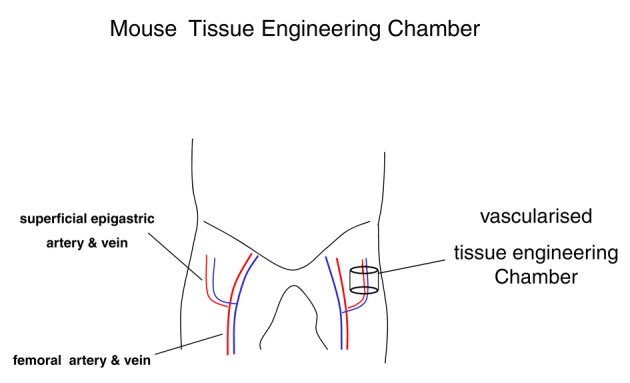
Mouse vascularised tissue engineering groin chamber model for growing primary human liposarcoma.

Tissue engineering chambers (n = 6) consisting of 5 mm of length of silicone laboratory tubing (3.35 mm internal diameter, 4.4 mL volume; Dow-Corning Corp., Midland, MI) were inserted into the groins of the mouse as previously described [23]. The animal was placed in a supine position, after the exposure of the groins of the mouse a longitudinal incision along the medial thigh was performed. The inguinal fat pad and the superficial epigastric artery and vein were dissected for a distance of 1 cm from their origin at the femoral vessels (Fig. 1).

The tissue engineering chamber was then placed around the vessels. The chamber was secured to the underlying muscle with a 10-0 nylon microsuture. The longitudinal slid and proximal aperture of the chamber was sealed with bone wax (Ethicon, Somerville, NJ). Care was taken to avoid occlusion of the vascular pedicle. Tumour fragments suspended in Matrigel® (BD Bioscience, Palo Alto, CA, US) were inserted into the chamber. At 37 °C the matrix solidifies and thus ensures that the cells remain in situ. Then the distal aperture of the chamber was also sealed, creating a semiseald chamber again taking care to not compromise the vascular patency. The construct was carefully returned to the dissected plan within the groin. The wound was closed in layers. The anaesthesia protocol was uneventful in all cases. All animals survived the surgical procedure and recovered well. After recovery animal behaviour returned to normal and remained unsuspicious. No wound complications were noted.

### Chamber harvest and specimen assessment

After an incubation of 3 weeks *in vivo* in which the animals were allowed to move freely and were fed a standard mouse chew *ad libitum* the animals were again anaesthetised. The wounds were explored and the chamber (tissue engineering chamber) was exposed. In the chamber the vascular patency was recorded. The vascular pedicle was cut outside the chamber and the chamber retrieved from the implantation site. The outside of the chamber was freed from surrounding fibrous capsular and the chamber reopened. The newly formed tissue was then carefully removed. Tissue specimens were photographed and the tissue was divided into two sections one for histological assessment the other one for electron microscopy. Tumour pathology was analyzed by histological and immunohistochemical staining.

### Histological assessment

Tumour samples were placed in 10% formaldehyde. A minimum of five individual sections of the primary tumour were assessed. The primary tumour and the xenotransplant counterpart were sectioned into 5 µm thick sections.

Histological sections were deparaffinised, rehydrated and stained with haematoxylin and eosin following standard procedures.

### Electronmicroscopy

Primary tumour and the xenotransplant specimens were fixed in 2% glutaraldehyde and embedded in Epon 812. For contrast uranyl acetate and lead citrate were added to the ultra thin sections. Samples were analysed, special focus was placed on differentiation criteria [1]. Microscopic features of the tumours such as cellularity, growth pattern, cytomorphology, vascularity, invasiveness, degree of differentiation, and necrosis were assessed.

## Results

Diagnosis of the primary human liposarcomas was confirmed by independent reference histology. Tumour diagnosis and classification were determined according to WHO guidelines [1].

### Histological and electronmicroscopic morphology of the primary tumours

**Figure 2:**
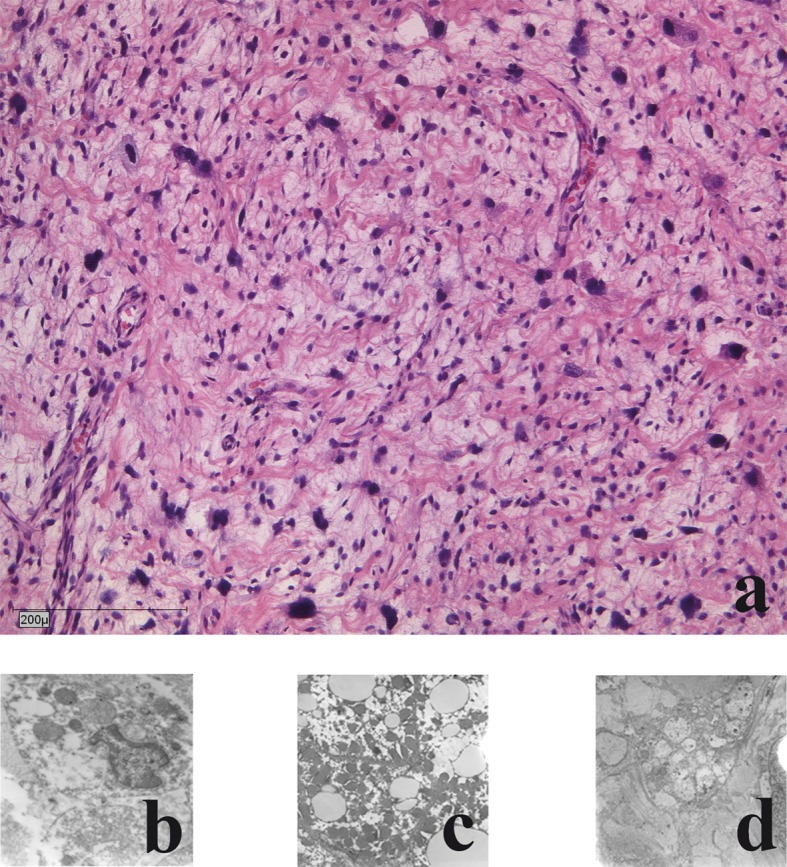
Histological and electron-microscopic morphology of the primary tumour: a) haematoxylin and eosin staining; b) Nuclear morphology demonstrates large atypical cell nuclei with heterochromic prominent nucleoli; c) Cytoplasm: few reticular endoplasmic reticuli with few dark elongated elliptical mitochondria; d) Lipid vacuoles: few with no accumulation of glycogen.

The liposarcomas showed a pleomorphic growth pattern with atypical mitoses and a marked increase in nuclear and cell changes. Bizarre pleomorphic lipoblasts were seen next to spindle shaped tumour cells, even some giant cells were found. Myxoid changes were rare and tumour necrosis was seen in 15 % of areas. Pancytokeratines (MNF116), EMA, smooth muscle actin, desmin, S100 and CD34 were negative (Fig. 2a).

Electron microscopic analysis showed malignant cells with mostly decreased rough endoplasmic reticulum and heterogeneity of cells and nuclei size and shape were present. Nuclear irregularities with multiple nucleoli were frequent. Nucleoli were increased in size and number. The sarcomatous cells had highly irregular nuclear profiles. Mitochondria were electron dark and elongated. Golgi complexes were present. No accumulation of glycogen was seen. Mitotic accumulation of lipid globules was a frequent finding. Few giant cells were present. Focally necrotic areas were seen (Fig. 2b-d).

### Histological and electronmicroscopical morphology of the xenotransplants

**Figure 3:**
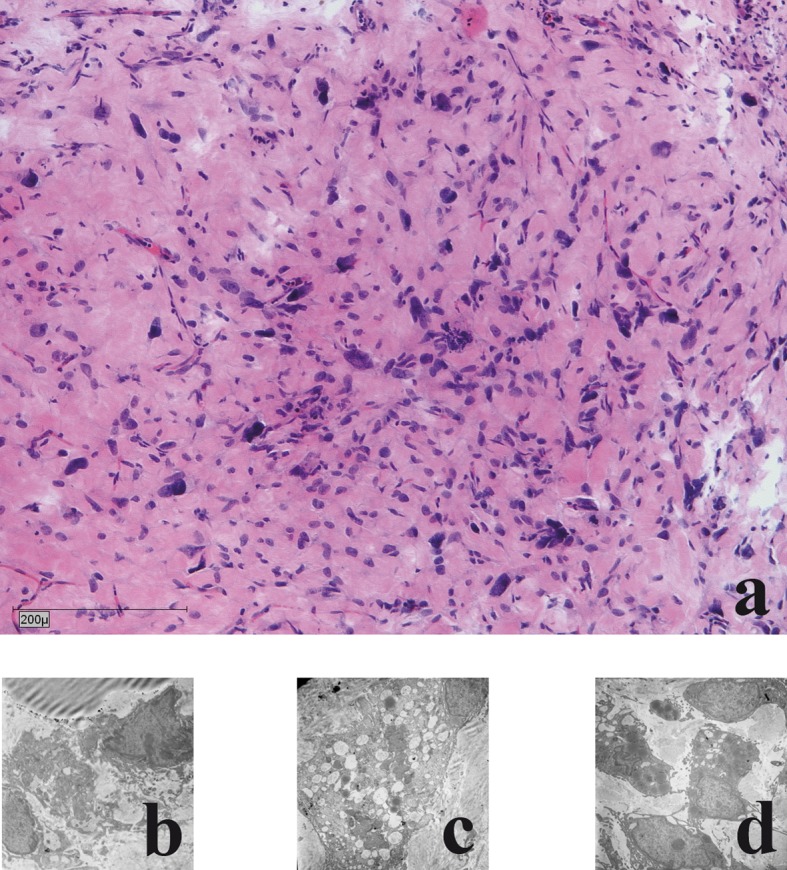
Histological and electronmicroscopic morphology of the xenograft: a) haematoxylin and eosin staining; b) Nuclear morphology demonstrates large oval atypical cell nuclei with heterochromic prominent nucleoli; c) Lipid vacuoles: few light and dark vacuoles with no accumulation of glycogen; d) Cytoplasm: few reticular endoplasmic reticuli with few dark elongated elliptical mitochondria.

In all cases (n = 6) the liposarcomas in the chamber model demonstrated a moderate, partial severe inflammation with giant cells was detectable. All cases showed circumjacent to the tumour, slight to moderate proliferations of fibroblasts (Fig 3a). Ultrastructural maintenance was good in the tumour grown in the chamber. Heterogeneity of tumour cells and nuclei was seen with irregularities of nuclei. Focally necrotic areas were seen. Heterogeneity of tumour cells and nuclei was seen with irregularities of nuclei and many small nuclei. Cell necrosis and cell disintegrations were common findings (in more than 50%) (Fig. 3b-d).

## Discussion

Animal models are indispensable tools for the study of liposarcomas as a paucity of clinical samples makes large-scale analysis of human samples challenging. Furthermore, the diversity of sarcoma subtypes complicates analyses of human sarcomas. Until recently, studies of sarcoma biology were limited to human cell lines and xenografted tumours. An in depth understanding of the tumour biology of these rare tumour entities is warranted to develop and improve sarcoma treatment thus demanding for a well characterized and reproducible *in vivo* tumour model. Xenotransplantation of tumour cells into immune deficient mice has been well recognised and is a useful experimental model in cancer research [15, 17-19, 24]. The subcutaneous skin fold chamber is an established animal model for malignant soft tissue tumours, but the high failure rate after transplantation as well as the instability regarding phenotype, differentiation and characteristics of the xenograft remains an unsolved problem [25-28]. A 50% take rate of human tumour xenograft has commonly been reported [25-28]. In a large comparative study of human sarcoma xenografts evaluating intraperitoneal and subcutaneous transplantation sites, Hajdu et al. found an overall take rate of 62%. But only 51% of all tumour specimens grew subcutaneously. 52% appeared less 13% better differentiated and only 35% resembled the primary tumour when compared to the human tumour resection specimen [18]. The intraperitoneal transplantation resulted in an even higher discordance of differentiation. It is well established that in the initial phase after transplantation cells are exposed to a hypoxic environment and rely on diffusion alone for nutrition [25-28]. Hence a diffusion barrier of more than 200 µm will hamper cell survival [29]. It can be hypothesised that this initial vulnerable phase can be accounted for the low graft take after xenotransplantation and may also promote the selection of more resistant hence more dedifferentiated tumour cells. The later is well reflected in the observation that on the one hand the subcutaneous xenograft model is more successful with high grade tumours and on the other hand that dedifferentiation of the tumour when compared to the human primary is frequently reported [17-19]. To overcome this obstacle in the present study, a vascularised chamber for cell transplantation was used.

The vascularised chamber was initially developed for cell transplantation in a tissue engineering setting at the Bernard O’Brien Institute of microsurgery [23]. It has been shown to successfully promote cells survival of thymus [30], cardiac muscle [31], myoblasts and various other tissues [32-34]. Centred around a vascular pedicle, the chamber allows for early and rapid neoangiogenesis supporting the nourishment of the transplanted cells and reducing the time period of hypoxia [23].

In the present, all of the xenotransplants into the groin chamber resulted in a survival of the transplanted liposarcomas. As expected from the tissue engineering experiments, an increased angiogenesis was also observed after xenotransplantation of tumour tissue within the groin chamber. All groin chambers displayed a dense functional newly formed vascular network. Tumour cells migrated within the matrix of the groin chamber and seemed to be evenly distributed throughout the chamber. A reduction of connective tissue within the tumour with an increased cell number after xenotransplantation has been described previously.

Immortalised tumour cell lines offer an attractive opportunity for cancer research. Their availability and standardised growth pattern make them suitable for routine testing, but their dedifferentiated nature and acquired additional genetic alterations during the immortalisation process may not reflect all aspects of the primary tumour [25-28]. Differences in phenotype of these cell lines often result from an arrest in differentiation process at specific stages [25-28]. Hence, it is consistently difficult to translate the behaviour of these cells in an experimental setting to a human soft tissue sarcoma *in vivo*. In addition, primary cell lines are known to acquire genetic alterations after tissue culture expansion. Therefore, in the present study we used tumour fragments transplanted directly after tumour resection to best be able to compare the growth pattern to the original human sarcoma. A potential disadvantage to the use of these tumour fragments includes the heterogeneity of the tumour fragment composition and therefore lack of standardisation.

No metastases were observed in any experiment. The short three week observation period may explain this. The tissue engineering chamber produced viable and good tumour growth and seems to be a favourable model to analyze early tumour implantation and tumour stromal interactions. It also offers the chance to better assess the tumour angiogenesis early after transplantation. On the other hand the space limitation with in the silicon chamber might hinder extensive tumour growth.

In conclusion, the vascularised tissue engineering chamber offers a suitable experimental sarcoma model. This model is valuable for sarcoma research because clinical samples are relatively rare and treatment regimens have not changed in decades for many sarcoma subtypes. The further development of models, including the vascularised tissue engineering chamber will continue to increase our understanding of soft tissue sarcoma biology. The susceptibility to hypoxia differs between the sarcoma subgroups and the type of soft tissue sarcoma, the vascularised tissue engineering chamber appears to have benefits in successful outcome of xenograft growth, in creating a more vital tumour that may be a more faithful model and on study of tumour stroma interaction and tumour angiogenesis
